# Maternal ancestry reveals cyclical aging of the mammary gland

**DOI:** 10.21203/rs.3.rs-4926839/v1

**Published:** 2024-11-21

**Authors:** Doris Germain, Thelma Mashaka, Mrittika Chattopadhyay, Dmitry Polushakov, Miguel Torres-Martin, Daniela Sia, Edmund Jenkins

**Affiliations:** Icahn School of Medicine at Mount Sinai; Icahn School of Medicine at Mount Sinai; Icahn School of Medicine at Mount Sinai; Icahn School of Medicine at Mount Sinai; Barcelona Institute of Science and Technology; Icahn School of Medicine at Mount Sinai; Icahn School of Medicine at Mount Sinai

## Abstract

We present provocative data that in addition to the expected progressive age-related involution, mammary gland aging can occur in a cyclical pattern and is dictated by maternal ancestry. In cyclical aging, mammary glands of 11 and 19 months old mice share genetic and proteomic signatures, which are enriched in breast cancer-related pathways, but are absent at 3 and 14 months. Since incidence of breast cancer shows a bimodal age distribution at 45 (~11m in mice) and 65 (~ 19m in mice), cyclical aging may contribute to these peaks of cancer susceptibility. Conversely, since the mammary glands at 3 and 14 months cluster together hierarchically, the cancer-associated peaks seem separated by a rejuvenation phase. Since cyclical aging is observed in mice with extended lifespan, these findings raise the possibility that if oncogenic mutations are avoided during the pro-oncogenic phases, through its rejuvenation phase, cyclical aging may impact multiple organs leading to extended longevity.

## Introduction

It is well established that chronological aging does not always correlate with biological aging, but the mechanism of this disconnection is not known. However, independently of whether it occurs slowly or more rapidly, the aging process is assumed to be linear and progressive.

Mitochondrial dysfunction is a hallmark of aging^1–5^. The mitochondria contain a small circular genome of 16kDa, which encodes only 13 proteins, while the mitochondria contain over 1000 proteins. Therefore, most mitochondrial proteins are encoded by nuclear genes. The canonical function of the mitochondria is the production of ATP via the activity of the electron transport chain (ETC). Some complexes of the ETC contain both mitochondrial and nuclear encoded subunits where the mitochondrial-encoded subunit is an essential core for the function of these complexes, and consequently affects global mitochondrial fitness. Therefore, the integrity of the mitochondria network requires a unique coordination between two genomes and rely on a robust mitochondria-nucleus communication. The sequence of the mitochondria genome however varies in the human population and this diversity is classified into a variety of mitochondria haplotypes^6–8^. The use of cybrids, which are cells that have the same nuclear genome but carry different mitochondria haplotypes, has demonstrated the profound impact of mitochondrial genetics on nuclear gene expression in multiple diseases^9–13^.

To investigate the impact of mitochondrial genetics *in vivo,* the Enriquez group used the fact that mitochondria DNA is maternally inherited to generated mice with the C57BL/6 nuclear genetic background (BL/6) but differ in mitochondria genomes, and carry either the C57BL/6 or NZB/OlaHsd (^C57^ or ^NZB^) background^14^. Since the number of nucleotide differences in the mitochondrial DNA between NZB/OlaHsd and BL/6C57 mice is within the same range as that observed between the European and African DNA haplotypes in the human population, these mice represent a model that reflects the natural diversity in mitochondrial genetics in humans^14^

The original analysis of these mice led to the discovery that the BL/6^nzb^ mice of both sexes live longer and show multiple differences in gene expression in the heart and the liver. In this study however, only two time points were investigated; young (12 weeks) and aged (2 years).

In the current study, we analyzed the aging of the mammary gland at 4 different ages that span the entire range of estrous cycle over aging^15^. We found that while the BL/6^C57^ females show the expected linear and progressive involution of the mammary gland over aging, the BL/6^nzb^ females show a cyclical aging pattern.

## Results

**Figure 1:**
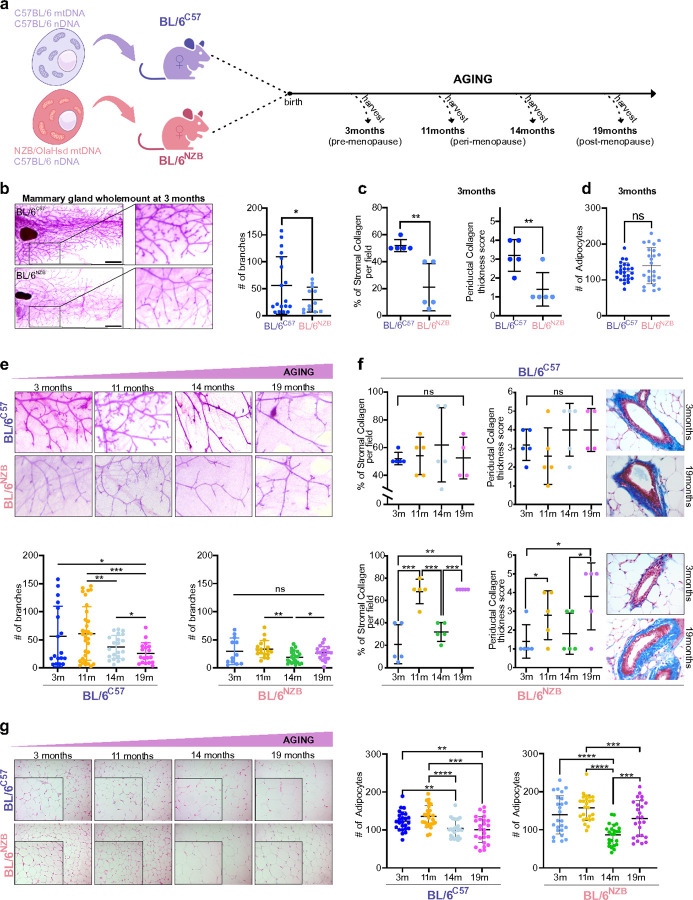
Mitochondrial DNA impacts the pattern of aging of the mammary gland. **(a)** Schematic of the experimental design. BL/6^C57^ and BL/6^nzb^ female mice were aged to the specified ages, and mammary glands were harvested for analysis at each time point (n=5 per time point for each genotype). Image created using Biorender.com. **(b)** Representative carmine-stained mammary gland whole mounts of BL/6^C57^ (top) and BL/6^nzb^ (bottom) showing mammary gland branching and quantification at baseline (3 months) **(c, d)** Quantification of **(c)** collagen by Masson Trichrome Staining and **(d)** adipocytes, highlighting baseline differences between the BL/6^C57^ and BL/6^nzb^ female mice. **(e)** Representative carmine-stained mammary gland whole mounts from BL/6^C57^ (top panel) and BL/6^nzb^ (bottom panel), illustrating age-related changes in mammary gland branching. Quantification of mammary gland branching in BL/6^C57^ (left) and BL/6^nzb^ (right) at 3, 11, 14 and 19 months is shown below. **(f)** Quantification and representative images of collagen (blue) by Masson’s trichrome staining of BL/6^C57^ (top panel) and BL/6^nzb^ (bottom panel) female mice. Two parameters were evaluated: (1) the percentage of stromal collagen per mammary gland and (2) periductal collagen thickness, assessed using an arbitrary scoring system ranging from 1 (thin/filamentous collagen) to 5 (thickest collagen). **(g)** Representative H&E images illustrating age-related changes in adipocyte content in the mammary glands of BL/6^C57^ (top panel) and BL/6^nzb^ (bottom panel) mice, followed by the quantification. Images were taken at 20X magnification. Data was analyzed using Welch’s t-test; (*P < 0.05, **P<0.01, ***P<0.001, ****P<0.0001).

We aged BL/6^C57^ and BL/6^nzb^ female mice at 3, 11, 14 and 19 months (Fig. 1a). We selected these time points to include 1) young adult (3 months) at the peak of their reproductive capacity, 2) 11 and 14 months where estrous cycles are still observed but start to fluctuate and progressively decrease in frequency and 3) 19 months, where estrous cycles are no longer observed^15^. Therefore, we reasoned that these ages were deemed to best recapitulate the involution of the breast over the menopausal status with aging in humans and include pre-menopause, peri-menopause and post-menopause status. We also selected to analyze; a) the number of mammary duct branches, b) stromal and peri-ductal collagen and c) adipocytes, based on the fact that these parameters have been reported to be altered during aging of the breast in humans^16–19^.

We first compared the mammary glands between genotypes at 3 months. We found that the BL/6^C57^ females have a significantly higher number of mammary branches, compared to the BL/6^nzb^ females (Fig. 1b). The amount of stromal and peri-ductal collagen was also higher in BL/6^C57^ females (Fig. 1c), however, the number of adipocytes was not significantly different between genotypes (Fig. 1d). These differences therefore suggest that mitochondrial genetics impact the development of the mammary gland at puberty and/or the development of the rudimentary mammary gland in utero, a possibility that will be investigated in the future. However, since the size, morphology and density of breasts in humans show a wide range of variation, we view these differences between genotypes at baseline as an indication that the mammary gland in mice also show variability. These differences in the mammary glands between mouse strains have been reported in early studies but are rarely considered in the current literature^20^

**Figure 2:**
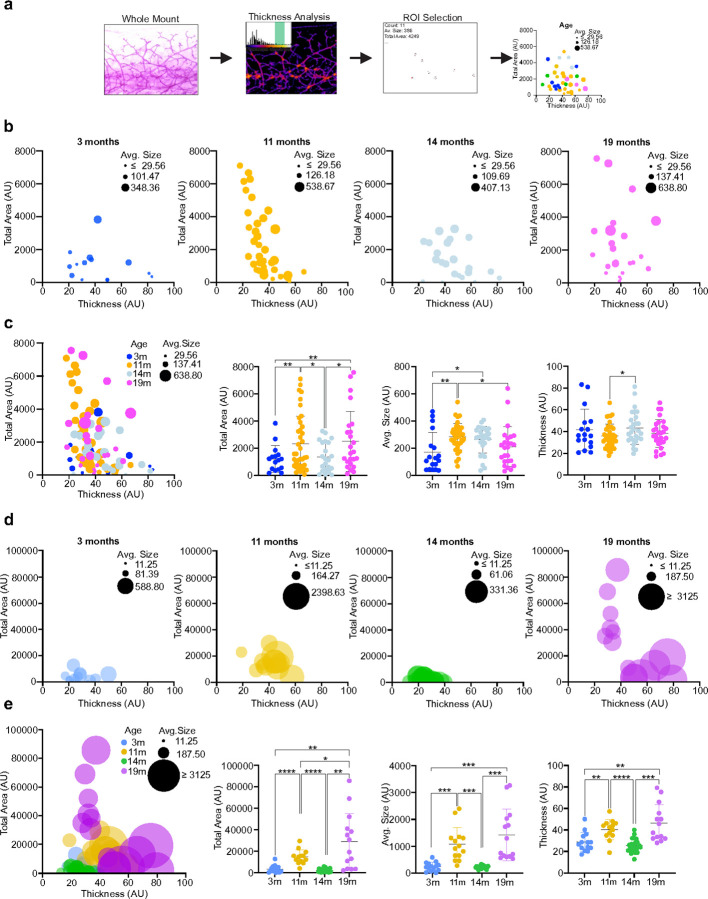
Susceptibility of the mammary gland to spontaneous intraductal hyperplasia. **(a)** A schematic of how spontaneous mammary intraductal hyperplasias were analyzed and quantified using an Image J plug-in. **(b,c)** Multi-variable graphs depicting the total area, average size, and thickness of spontaneous intraductal hyperplasia in BL6/^C57^ mice at (b) individual time points and (c) combined time points. Quantification highlights the differences observed across different age groups. **(d,e)** Multi-variable graphs depicting the total area, average size, and thickness of spontaneous intraductal hyperplasia in BL6/^nzb^ mice at (d) individual time points and (e) combined time points and quantification highlighting differences observed across different age groups.

We then compared the mammary glands at different ages within each genotype. In the BL/6^C57^ females, we found that the number of mammary branches decreases gradually with age (Fig. 1e), while in BL/6^nzb^ females, no difference was observed between 3 and 11 months, a decrease was observed between 11 and 14 months followed by an increased at 19 months (Fig. 1e). No difference in stromal and periductal collagen over aging was observed in BL/6^C57^ females (Fig. 1f). However, a highly significant fluctuation pattern (increased level followed by decreased level followed by increased level) in stromal collagen staining was observed in BL/6^nzb^ females over aging (Fig. 1f) and the same trend was also observed for peri-ductal collagen (Fig. 1f). Finally, the number of adipocytes was found to gradually decrease in BL/6^C57^ females over aging (Fig. 1g), while the pattern of a decrease between 11 and 14 months followed by an increase at 19 months was observed in BL/6^nzb^ females (Fig. 1g). To complete our morphological analysis of the mammary gland over aging, we then used digital imaging of the mammary gland to measure region of interest showing increased intra-ductal thickness (Fig. 2a). Using three independent measures; total area, average size of the area and thickness, we found that no consistent pattern between measurements was observed over aging in the BL/6^C57^ females (Fig. 2b, c). In contrast in the BL/6^nzb^ females, a pattern of increase at 11 months followed by a decrease at 14 months followed by an increased at 19 months was observed using all three measurements (Fig. 2d, e). Furthermore, the scale of total area (size + thickness) was 10-fold higher in BL/6^nzb^ compared to BL/6^C57^ females (Fig. 2c, e). Collectively, this array of diverse and independent analyses of the mammary glands (numbers of branches, collagen, adipocytes and ductal thickness) suggests that while the mammary glands of the BL/6^C57^ females show the expected pattern of gradual regression over aging, in the BL/6^nzb^ females, the pattern of aging of the mammary glands shows a recurrent cyclical pattern, where the mammary glands at 3 and 14 months behave more similarly and the 11 and 19 months behave more similarly.

**Figure 3:**
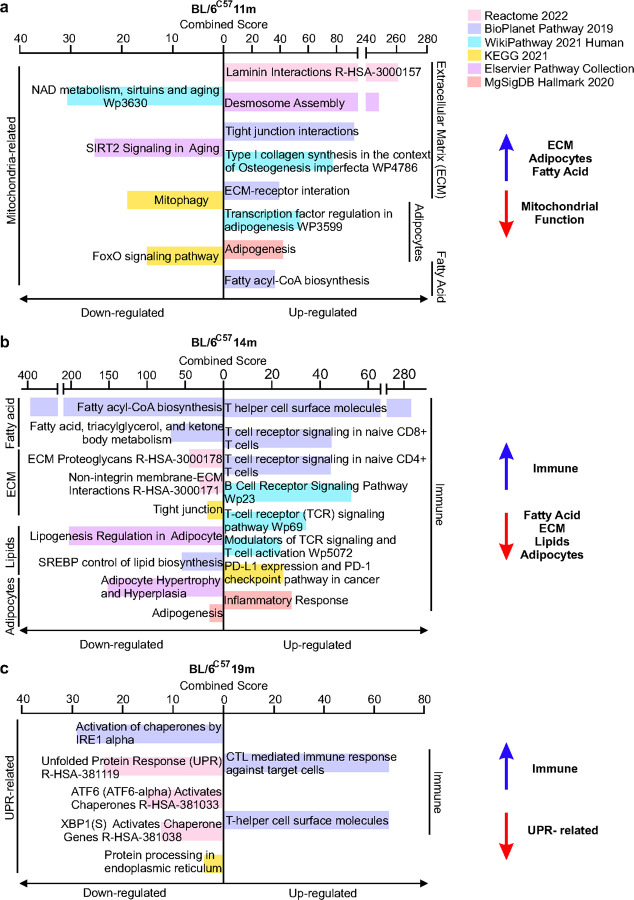
Bulk RNA Seq analysis of the mammary gland in BL/6^C57^ females reveals decline in mitochondrial function and increase immune modulation with age. Top pathways identified by gene set enrichment analysis of differentially expressed genes in BL/6^C57^ female mice comparing **(a)** 11m relative to 3m, **(b)** 14m relative to 11m, and **(c)** 19m relative to 14m. Analysis was performed using the Enrichr pathway analysis platform.

Intrigued by this recurrent cyclical pattern of aging in BL/6^nzb^ females, we first performed bulk RNAseq on the mammary glands at all ages and in both genotypes. This analysis revealed that in BL/6^C57^ females, an increase in ECM and adipocytes remodeling and a decrease in mitochondrial function are among the pathways most differentially regulated in early aging (11 months versus 3 months) (Fig. 3a). Altered immune related pathways were observed at a later stage of aging (14 months versus 11 months and 14 months versus 19 months). These findings reinforce previous aging studies in general and are consistent with age-related involution of the breast^19,21–23^. Further, they also overlap with the findings from a recent study from the Brugge group, where single cell RNA seq was performed on the mammary glands of 3 and 14 months old female mice, where a decline in mitochondrial function was reported^21^. Therefore, we repeated the analysis using 3 and 14 months BL/6^C57^ females rather than 3 and 11 months. This analysis confirmed a decline in mitochondrial function (Extended data 1). Therefore, since our study includes an additional time point and mitochondrial function decline is observed between 3 and 11 months but not when 11 and 14 months are compared, we conclude that mitochondrial function is an early event in the aging of the mammary gland in BL/6^C57^ female mice.

**Figure 4:**
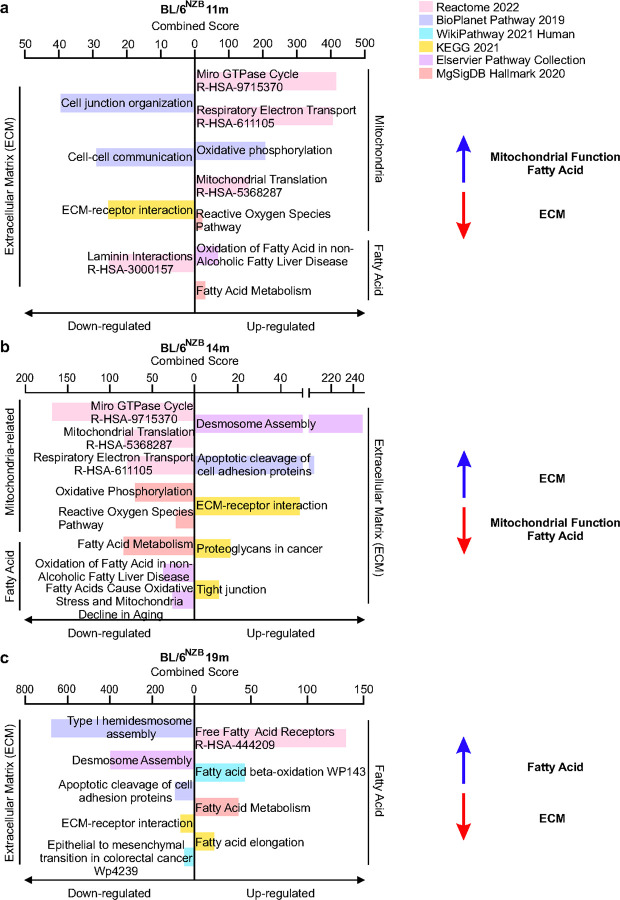
Bulk RNA Seq analysis of the mammary gland in BL/6^nzb^ females reveals cyclical modulation of mitochondrial function and ECM i over aging. Top pathways identified by gene set enrichment analysis of differentially expressed genes in BL/6^nzb^ female mice comparing **(a)** 11m relative to 3m, **(b)** 14m relative to 11m, and **(c)** 19m relative to 14m. Analysis was performed using the Enrichr pathway analysis platform.

The same RNAseq analysis in BL/6^nzb^ females revealed the opposite trend with an increase in mitochondrial function and a decrease in ECM and adipocytes related pathways between 3 and 11 months (Fig. 4a), a reversal of this pattern between 11 and 14 (Fig. 4b) and again a reversal between 14 and 19 months (Fig. 4c). The bulk RNA seq analysis therefore reinforced the notion of a cyclical aging pattern of the mammary gland in the BL/6^nzb^ females.

To gain more refined and quantitative insights into the potential cyclical aging pattern in BL/6^nzb^ females, we performed two additional analyses. In the process of harvesting the mammary glands at different ages, we collected a fraction of the mammary glands for decellularization and analysis of the extracellular matrix. Additionally, we digested a fraction of the mammary glands and eliminated the fibroblasts with the intent to generate primary epithelial mammary cells (PMEC) for future pathway analysis. We initially aimed at testing mitochondrial function by Seahorse on PMEC at different ages but discovered that these cells do not survive the plating process. Therefore, in light of the unexpected pattern of aging observed in the BL/6^nzb^ females, we decided to use the decellularized mammary gland to perform proteomics due to the strong ECM signaling pathways identified by bulk RNAseq and use the purified primary mammary epithelial cells for single cell RNAseq analysis.

**Figure 5:**
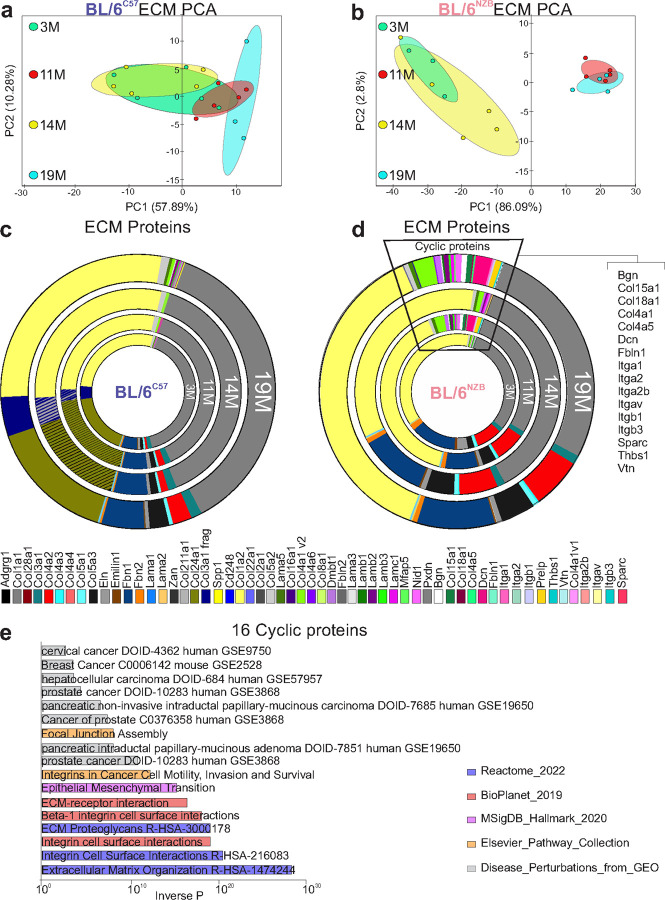
Proteomics of decellularized mammary glands identified 16 cycling proteins in BL/6^nzb^ females. **a-b)** Principal component of proteomic analysis of ECM derived from decellularized mammary glands at 3,11, 14 and 19 months in BL/6^C57^ **(a)** and BL/6^nzb^ **(b)** females. **c-d)** Pie graphs of proportional distribution of indicated ECM proteins according to age in BL/6^C57^ **(c)** and BL/6^nzb^ females **(d)**. Triangle indicates the 16 proteins found specifically up regulated at 11 and 19 months in BL/6^nzb^ females. **e)** Pathway analysis related to the 16 cycling ECM proteins identified in BL/6^nzb^ females.

Principal Component Analysis (PCA) of the proteomics dataset from the decellularized mammary glands at each time points in the BL/6^C57^ female mice showed no specific clustering between age groups (Fig. 5a). In contrast, in BL/6^nzb^ females, a strong clustering of ECM proteins was observed between the 3 and 14 months on one hand, and on the other hand, a strong clustering between the ECM proteins derived from mammary glands at 11 and 19 months was observed (Fig. 5b). The result of the PCA analysis was confirmed by analyzing individual extracellular matrix (ECM) proteins. We found that the distribution was largely similar between age groups in the BL/6^C57^ females (Fig. 5c). Interestingly, the ECM of the BL/6^C57^ females at all ages, contain some sub-types of collagen that were not found in the ECM of the BL/6^nzb^ females (Fig. 5c, olive green) suggesting that these specific proteins may be associated with linear aging.

In contrast to the BL/6^C57^ mice, PCA analysis of ECM proteins in the BL/6^nzb^ females showed a strong clustering of proteins at 3 months and 14 months as well as those derived from 11 months and 19 months old females (Fig. 5b). Further, 16 ECM proteins were found to be unique to the mammary glands of BL/6^nzb^ females but also specific to 11 and 19 months and not found at 3 and 14 months, suggesting that the expression of these proteins follow a cyclical pattern of expression over aging (Fig. 5d). To further investigate the potential role of these proteins, we performed pathway enrichment analysis related to the combined expression of these 16 cyclical proteins. We found pathways related to cancer such as Cancer Cell Motility, Invasion and Survival, Akt signaling and mesenchymal transition (Fig. 5e). This result suggests that the extracellular matrix of BL/6^nzb^ females at 11 months and 19 months may be more permissive to the proliferation and invasion of cancer cells.

**Figure 6:**
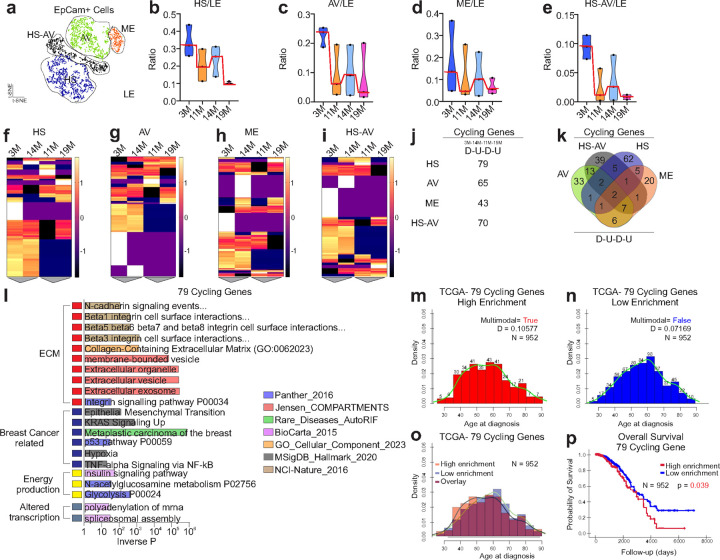
Single cell RNAseq analysis shows cyclical fluctuation in the number of individual cell populations and their transcriptomes. **a)**t-SNE clustergram of single cell RNAseq data from 12 mice. HS= Hormone sensitive, HS-AV= hormone sensitive alveolar cells, AV= alveolar cells, ME= myoepithelial cells. b-e) Ration of HS cells **(b)** AV cells **(c)** ME cells **(d)** or HS-AV cells **(e)** vs total luminal epithelial (LE) cells in 3M, 11M, 14M, and 19M mice (n=3 mice per time point). **f-i)**Heatmap clustergrams of differential gene expression data generates by comparing HS cells **(f)**, AV cells **(g)** ME cells **(h)**, or HS-AV **(i)** epithelial cells. **j)** Number of genes that in each cell population whose expression patter cycles down (D) then up (U) over the 3M, 11M, 14M, and 19M time points. **k)** Venn diagram of the cycling genes in panel J. **l)** Gene Set Enrichment analysis of the 79 cycling genes identified in the HS cell population.Histograms of patient age at diagnosis for patients highly enriched **(m)** or low enriched **(n)** cyclic gene signature. **o)**Overlay of panels M and N. **p)** kaplan-meier analysis of patients with high enrichment vs low enrichment of the 79 cyclic gene signature. Modality was determined using Hartigan’s dip test and the LaplacesDemon package in R. Kaplan-Meier estimates of overall survival of high enrichment score for the 79-gene signature compared to low enrichment score.

We then performed single cells RNAseq analysis on primary epithelial cells derived from BL/6^nzb^ females. This approach was taken to enrich for epithelium while reducing the number of fibroblasts, which may result in the elimination of other cell types of the stroma including the immune cells. Four different types of mammary epithelial cells were identified; the luminal hormonal sensitive cells (HS), the luminal Alveolar cells (AV), the hormonal/alveolar population (HS-AV) and the myoepithelial cells (ME) (Fig. 6a). We used the total number of luminal epithelial cells (LE) as a common denominator to normalize the content of each cellular sub-types. We found that in each case, the ratios of HS/LE (Fig. 6b), AV/LE (Fig. 6c), ME/LE (Fig. 6d) and HS-AV/LE (Fig. 6e) show a cyclical pattern with a lower ratio at 11 months and 19 months and higher ratio at 3 months and 14 months. We then analyzed the cell-type specific transcriptome of each cell types according to age. Remarkably, we found that in each case, the gene expression profiles of cells at 11 months and 19 months hierarchically cluster together, while the transcriptomes at 3 months and 14 months hierarchically cluster together (Fig. 6f–i). This finding indicates that the transcriptional program of individual cell types in the mammary gland also follows a cyclical pattern. We then search for genes that adopt the cyclical pattern of expression over aging in each cell types and found 79 genes in the luminal HS cells, 65 genes in the AV cells, 43 genes in the ME cells and 70 in the mixed lineage of HS-AV cells (Fig. 6j). Comparison of these gene signatures revealed that they are largely cell-type specific as only few genes are shared between signatures (Fig. 6k).

To interrogate the role of the cyclic genes, we perform pathway analysis of the 79 cyclic genes specifically expressed in the luminal HS cells and found that as observed for the cyclic ECM proteins, these pathways are enriched in breast cancer-related pathways such as epithelial mesenchymal transition, p53, hypoxia and Ras signaling (Fig. 6l, Extended data 2). This result further supports the notion that cyclical aging is associated with pro-tumorigenic phases at 11 months and 19 months. Pathway analyses of the other cell specific cycling signatures reveal some cancer related pathways, although not as consistently for the HS cells (Extended data 3, 4, 5).

Since cyclical aging is observed in BL/6^nzb^ females but not in the BL/6^C57^ females and these mice only differ in mitochondrial genetics, by extrapolation to the human population, our data suggest that only a subset of women carrying specific mitochondrial haplotypes may experience a cyclical aging pattern of the breast. Further, our data suggest that these women may be more prone to develop breast cancer at the age of 45 and 65 (the equivalent of 11 months and 19 months in mice). This possibility is supported by the fact that the incidence of breast cancer shows a bimodal age distribution at 45 and 65^24–26^. To test if cyclical aging may happen in humans, we assess the frequency at which the 79 cyclical genes signature, derived from normal mammary gland in our mouse model, is observed in normal breast tissues. We identified a database of RNAseq data from 204 normal breast samples derived from reduction mammoplasty. We found that the 79 cyclical genes signature of HS cells is observed in 12 out 204 cases (9.7%). However, the age was not available for the 204 cases such that association with age was not possible. A frequency of 9.7% is within the range that is expected since only women having reduction mammoplasty within the equivalent of 11 months and 19 months phases of cyclical aging in mice are expected to show this signature and the life time risk of breast cancer in all women is ~12%. Next, we reasoned that if cyclical aging is associated with increased risk of breast cancer, the cyclical gene signatures should be expressed in breast cancers and enriched in patients around 45 and 65 years of age. We therefore analyzed data from 952 primary breast cancers in the TCGA breast cancer dataset. In this dataset, 82% of breast cancers are positive for the estrogen receptor (ERa), which makes it ideal for the analysis of the 79 cyclical genes derived from the HS cells. We performed enrichment score for the 79 cyclical genes signature and divided the cohort into either High enrichment score (1/3 of patients) or Low enrichment score (2/3 of patients). Using this cut-off, we then perform distribution plot according to age of diagnosis and found that the distribution of patients carrying High enrichment score follows a bimodal distribution with peaks at 45 and 55–65 (Fig. 6m), while the age distribution of patients classified within Low enrichment score show a unimodal distribution with a peak at 65 (Fig. 6n). Overlay of the density plots highlighted an earlier age of diagnose for patients classified in the High score category (Fig. 6o), an observation confirmed independently using a comparison of clinical characteristics between patients with high and low enrichment score of the 79 genes signature. Patients with High enrichment score were more likely to be younger (< or equal to 43 y.o.) (Extended data 6).

Additionally, since this 79 genes signature is associated with oncogenic pathways, we tested if the presence of the signature correlates with overall survival and found that patients with High enrichment score show significantly poorer overall survival (Fig. 6p). However, no association with metastases or breast cancer sub-types were found. We also tested TCGA breast cancer dataset for cyclical genes signatures found in other cell types and found they are not associated with survival.

## Discussion

Some of the most significant questions regarding aging are; how long will we live? What is the reason for the apparent disconnect between chronological aging and biological aging at least in some individuals? Can aging be delayed? What is the connection between aging and disease? The discovery of cyclical aging described in the current study may provide a novel and unexpected insight toward answering these fundamental questions.

In terms of the connection between aging and disease, our study offers a potential novel mechanism contributing to the bimodal age distribution of breast cancer, where two peaks of incidence are observed, one at 45 year of age and another at 65^24–26^. Breast cancers are classified based on the expression of three receptors, including the estrogen receptor alpha (ERα). The incidence of breast cancers in younger women, peaking at 45, tend to be ERα negative, while breast cancers in post-menopausal women, peaking at the age of 65, tend to be positive for the ERa^25^. Clinically therefore, the bimodal age distribution of breast cancers is defined uniquely by the ERα status. We recently reported that this age-dependent correlation between breast cancer and ERα status can be recapitulated in mice and proposed that the differential ability of luminal (ERα positive) and basal (ERα negative) epithelial cells to cope with organellar stress maybe at the origin of this observation^27^. However, while the susceptibility to transformation of different sub-types of epithelial cells is likely to play a role in the bimodal distribution of breast cancer, it does not explain why the peaks happen at 45 and 65 and not at 30 and 75 for instance. The results presented here suggest that the underlying reason for the peaks at 45 and 65 is that in a sub-set of women carrying specific mitochondrial haplotypes, the breasts at these ages share several features both in term of composition of the extracellular matrix and epithelial transcriptomes and these features increase the susceptibility to oncogenic transformation. Based on the 79 cyclical genes signature, our preliminary estimate less than 9.7% of women may show cyclical aging of the breast. However, several factors limit the interpretation of this estimate. First, the number of cases available in the normal breast RNAseq dataset is only 204 cases such that the representation of the diversity of mitochondrial haplotypes in this cohort is limited. Second, some women may have cyclical aging but had their mammoplasty performed at an age when the cyclical genes signature is not expressed.

Currently, clinical management of breast cancer is based on their classification by receptors status and the therapies are designed accordingly. The discovery of cyclical aging of the mammary gland raises the possibility to develop novel therapies aimed at targeting the features that are shared between peaks of susceptibility and combine such therapy with the current sub-type specific therapies.

Equally important is the finding that cyclical aging appears to involve a rejuvenation phase at 14 months, in between the two cancer-susceptibility phases at 11 months and 19 months. The suggestion of rejuvenation phase is based on the observation that at both the ECM proteomic and transcriptomic levels, the mammary glands of the 14 months old BL/6^nzb^ females are most similar to the mammary glands of the 3 months old BL/6^nzb^ females. What events and signaling pathways initiate this transition from the 11 months to the 14 months and its reversal between 14 and 19 months is a critical avenue of future research.

The discovery of the rejuvenation phase addresses directly the global interest into whether aging can be delayed using interventions such as diet and exercise. In cyclical aging, the rejuvenation phase is part of a normal physiological process that does not require any intervention.

It is tempting to speculate that cyclical aging may represent a novel example of chronobiology, which is defined as biological processes that repeat themselves over a specific period of time. The best-known example of chronobiology is the circadian rhythm over a period of 24 hours but others include diurnal, ultradian and infradian cycles, indicating that chronobiology affects all aspects of life from plant to humans^28–31^. Interestingly, disruption of circadian cycles is associated with aging^32^. Further, mitochondrial biology is intimately linked to circadian rhythm and novel connections between these two processes are continuously being discovered^33–37^. Dysfunctions of circadian rhythm and mitochondrial biology are both linked to aging^33,35,38^. Since our mouse model only differ in mitochondria, cyclical aging may be an additional link between the mitochondria and chronobiological processes.

Another important question that the discovery of cyclical aging raises is whether it also takes place in other organs in BL/6^nzb^ mice and whether it affects males as well. The fact that BL/6^nzb^ mice of both sexes were first reported to show extended life-span^14^ it is also tempting to speculate that the rejuvenation phase of cyclical aging may contribute to the delayed aging of multiple organs and contributes to the extended lifespan in these mice. We will actively seek to investigate these possibilities in the future.

During the preparation of this manuscript, a study of 108 participants reported a non-linear pattern of aging using a multi-omics analysis of blood, stool, skin, oral and nasal microbiome over aging (Shen et al. Nature Aging, Aug 14, 2024, Online access). The authors concluded that *“substantial dysregulation occurring at two major periods occurring at approximately 44 years and 60 years of chronological age”*. The conclusion of their study overlaps with our findings in two fundamental ways; first, the observation of not linear aging and second, that alterations in the aging pattern are observed around the ages of 40 and 60. However, since their study uses multiple sources of material including blood, which may reflect the aging of multiple organs, it is currently not possible to distinguish whether their observations reflect cyclical aging of particular organs or tissues. Further, the diversity of mitochondrial haplotypes represented in the population of participants used in their study is not known. If the fluctuation they describe is cyclical aging, the fact that they were able to detect these fluctuations within 108 participants would suggest either that this population is enriched in individual carrying mitochondrial haplotypes that confer cyclical aging or conversely, that our estimation of 9.7% is an underestimate due to an enrichment of mitochondrial haplotypes that confer linear aging among the 204 mammoplasty patients we used to make our estimate. Alternatively, their study raises the possibility that cyclical aging may occur in different organs in different individual and may not be uniform across organs or multiple types of cyclical aging exist. All these possibilities remain to be tested in the future.

**Figure 7:**
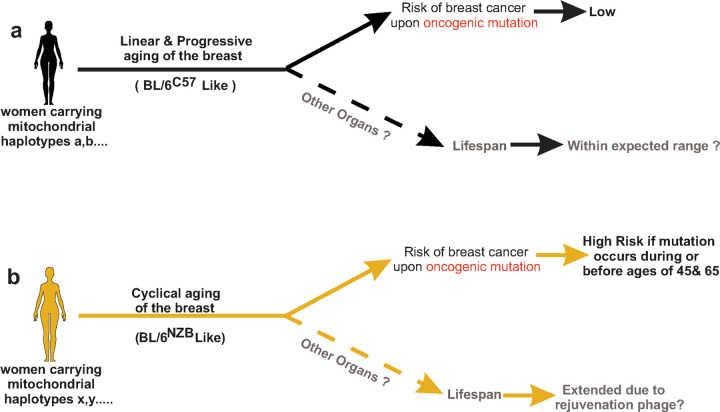
Graphical summary of the hypothesis of the outcome in women carrying certain mitochondrial haplotypes **(a)**experiencing linear and progressive involution of the breast over aging and **b)**in women carrying other mitochondrial haplotypes, where cyclical aging is observed. See discussion for details.

In summary, our results suggest that at least two distinct patterns of aging of the breast may exist and that maternal ancestry, through inheritance of mitochondria, determines which pattern is adopted. The results presented here support the hypothesis that in hypothetical women carrying certain mitochondrial haplotypes (Fig. 7a), the expected linear and progressive involution of the breast is observed over aging. In this setting, if an oncogenic mutation is acquired in the breast, the risk of developing breast cancer is lower. Further, if the same progressive and linear pattern of aging observed in the breast applies to other organs, the expectation is that these women will have a life span within the expected range (Fig. 7a). In hypothetical women carrying other mitochondrial haplotypes, cyclical aging is observed (Fig. 7b). In this case, cyclical aging is associated with both pros and cons. On one hand, if an oncogenic mutation is acquired in the breast around or during the cancer-susceptibility phases peaking at 45 and 65, since both the ECM and epithelial transcriptomic are pro-tumorigenic, the risk of developing breast cancer would be higher. On the other hand, we hypothesize that if cyclical aging is affecting multiple organs, but disease-causing mutations are not acquired, the rejuvenation phase of cyclical aging would promote extended longevity (Fig. 7b).

The discovery of cyclical aging therefore raises multiple questions and represent an exciting new area of aging research that holds the promise of having wide implications toward our understanding of aging, longevity and the link between aging and diseases.

## Materials and Methods

### Mice

All mouse experiments were conducted per an approved protocol by the Institutional Animal Care and Use Committee (IACUC). BL/6C57 and BL/6NZB mice were originally generated and kindly provided by Dr. José Antonio Enríquez in the C57BL/6JOlaHsd background. The mice were housed in vivariums at the Icahn School of Medicine at Mount Sinai with *ad libitum* access to food and water. BL/6C57 and BL/6NZB female mice were aged and harvested at specified time points (n = 5 per timepoint for each genotype), and mammary glands were harvested and stored appropriately for subsequent analysis.

### Wholemounts

Mouse abdominal mammary glands were harvested, mounted on microscope slides, and air-dried briefly. The glands were then fixed in Carnoy’s fixative (75% Ethanol, 25% glacial acetic acid) overnight. Subsequently, they were washed in 70% ethanol for 15 minutes, followed by a 5-minute water rinse, and stained overnight in carmine alum solution (1g carmine (Sigma C1022) and 2.5g aluminum potassium sulfate (Sigma A7167) in 500ml dH_2_O) according to standard protocol. After staining, the carmine-stained whole mounts were rinsed in MilliQ-filtered water for 5 minutes and dehydrated through an alcohol series (70%, 95%,100% EtOH, 15 minutes for each step). The glands were cleared in xylene and mounted using a Permount mounting medium. Finally, the whole mounts were imaged using a Zeiss Stemi 300 microscope.

### Masson Trichome Stain and H&E

Mammary glands were fixed in 10% formalin and subsequently processed and paraffin-embedded for sectioning by the Mt Sinai Biorepository Core Facility. Formalin Fixed Paraffin Embedded (FFPE) sections were prepared and stained for hematoxylin and eosin (H&E) according to standard protocol. Additional unstained slides were processed for collagen using the Fisher Scientific Epredia Richard-Allan Scientific Masson Trichrome Kit following the manufacturer’s instructions. Stained slides were imaged using a Zeiss AX10 Microscope. Quantification of collagen content was performed by calculating the percentage of stromal collagen per mammary gland and assessing periductal thickness using an arbitrary scoring system ranging from 1(thin/filamentous collagen) to 5 (thick collagen). This scoring was conducted by a blinded observer.

### Mammary gland decellularization

Freshly dissected mammary glands were decellularized by immersion in 1% SDS in TBS containing penicillin/streptomycin and DNase (1U/mL) in a 15 mL Falcon tube. The tubes were shaken at room temperature for 48 hours with 6 complete changes of the decellularization solution. Subsequently, the glands underwent an additional 48-hour period with 6 complete changes of water, (as described by Jenkins et.al., 2022). After decellularization, the mammary glands were frozen in 10% DMSO in TBS to preserve the tissue integrity before the samples were sent for proteomics analysis.

### Proteomics: Protein digestion

Decellularized mammary glands were resuspended in 50 uL of 8M Urea, 50mM EPPS and treated with DL-dithiothreitol (5mM final concentration) for 2 hours at 37°C with shaking (1400 rpm) on a Thermomixer (Thermo Fisher). Free cysteine residues were alkylated with 2-iodoacetamide (10mM final concentration) for 30 minutes at 25°C in the dark. Urea was diluted to 2M with Ammonium Bicarbonate (ABC) 100mM and PGNase added for 2 hours 37°C with shaking (1400 rpm). LysC (1:100 enzyme:protein) was added, followed by incubation for 2h at 37°C at 1400 rpm. Finally, trypsin (1:100 enzyme:protein) was added, followed by overnight incubation at 37°C at 1400 rpm. After the overnight incubation, the digest was acidified to pH<3 with the addition of 50% trifluoroacetic acid (TFA), and the peptides were desalted on 3-plug C18 (3M Empore^™^ high performance extraction disks) stage tips. Briefly, the stage tips were conditioned by sequential addition of i) 100 μL Methanol, ii) 100 μL 70% Acetonitrile (ACN)/0.1% TFA, iii) 100 μL 0.1% TFA twice. Following conditioning, the acidified peptide digest was loaded onto the stage tip. The stationary phase was washed once with 100 μL of 0.1% Formic Acid (FA). Finally, samples were eluted using 50μL of 70% ACN/0.1% FA twice. Eluted peptides were dried under vacuum followed by reconstitution in 12 μL of 0.1% FA, sonication and transfer to an autosampler vial. Peptide yield was quantified by NanoDrop (Biotek Synergy H1).

### MS analyses

Peptides were separated on a 50 cm column composed of C18 stationary phase (Thermo Fisher ES903) using a gradient from 0.5–25% buffer B over 100 minutes, to 50% over 15 minutes, to 90% in 5 minutes (buffer A: 0.1% FA in HPLC grade water; buffer B: 99.9% ACN, 0.1% FA) with a flow rate of 300nL/min using a nanoAcquity Hplc system (Waters). MS data were acquired on a Eclipse mass spectrometer (Thermo Fisher Scientific) using a data-independent acquisition (DIA) method. The method consisted of one MS1 scan, 400000 AGC target, maximum injection time of 50 msec, scan range of 380–985 m/z and a resolution of 120K. Fragment ions were analyzed in 60 DIA windows, maximum injection time of 40 msec, 100000 AGC target at a resolution of 15K.

### DIA Data Analysis

Raw data files were processed using Spectronaut version 18.5 (Biognosys) and searched with the PULSAR search engine with a mouse UniProt protein database downloaded on 2022/09/26 (94,765 entries). cysteine carbamidomethylation was specified as fixed modifications, while Methionine oxidation, acetylation of the protein N-terminus and Deamidation (NQ) were set as variable modification. A maximum of two trypsin missed cleavages were permitted. Searches used a reversed sequence decoy strategy to control peptide false discovery rate (FDR) and 1% FDR was set as threshold for identification. Unpaired t-test was used to calculate p-value in differential analysis, volcano plot was generated based on log2FC and q-value (multiple testing corrected p-value using Benjamini-Hochberg method). A q-value of < 0.05 was considered the statistically significant cut-off.

### Bulk RNA sequencing

Mammary glands of BL/6^C57^ and BL/6^nzb^ females at 3, 11, 14 and 16 months of age were flash frozen on liquid nitrogen and sent to Azenta for bulk RNA sequencing. The RNA sequencing data was analyzed using BioJupies, an interactive online platform that automates the generation of principal component analysis, hierarchical clustering, and differential gene expression analysis. Gene Set Enrichment Analysis was performed using Enrichr, focusing on the top differentially expressed genes and identifying enriched biological pathways associated with the dataset.

### Single Cell RNA Sequencing and Analysis

Freshly isolated snap frozen Inguinal mammary glands were submitted to GENEWIZ, LLC, NJ, USA for nuclei isolation and GEM (Gel Bead-based Emulsion) single cell RNA seq analysis on the 10x Genomics platform.Analysis was performed using the Cellranger (10xGenomics) count pipeline. Cell populations were identified using the Loupe Browser (v8.0.0, 10x Genomics) by the following features: Mfge8, Trf, Csn3, Wfdc18, Ltf (Luminal Alveolar (AV) cells), Prlr, Pgr, Esr1, Cited1, Prom1 (Luminal Hormone Sensitive (HS) cells), Krt17, Krt14, and Krt5 (Myoepithelial cells). Proliferating cells were identified by the combined feature sum expression of Cenpe, Ccna2, Ccnb2, Mcm6, Ccnf, and Bud1 as described^21^. Regulon expressing cells were identified by the combined feature sum expression of Bclaf1, Cux1, E2f1, E2f4, Esr1, Foxm1, Gtf2b, Max, Myc, Nfya, Nr4a1, Nrf1, Smarca4, and Taf1 as described^39^. The PPBC-like sub-population was identified among total barcodes by co-expression of both the Proliferating Cell and Regulon feature list. A filter cutoff to identify this population was set for half of the maximum Log2 feature sum for both feature lists. Differential gene expression for the Luminal-HS population was performed using the Loupe browser (v8.0.0) for each sample and assembled into a single .csv file that was uploaded to the Biojupies platform(https://doi.org/10.1016/j.cels.2018.10.007) to produce hierarchically clustered heatmaps using clustergrammer (developed by the Ma’ayan Laboratory at Mt. Sinai). The top 40 most differentially expressed genes between Bl/6 C57 and Bl/6 NZB LacD21 samples were then entered in EnrichR^40,41^ (https://doi.org/10.1002/cpz1.90) for pathway analysis across multiple databases.

### Survival prediction in the TCGA dataset

RNAseq normalized counts from the BRCA dataset were obtained from the UCSC Xena Hub: (https://tcga.xenahubs.net/download/TCGA.BRCA.sampleMap/HiSeqV2.gz). Clinical data were downloaded from the same resource at: (https://tcga.xenahubs.net/download/TCGA.BRCA.sampleMap/BRCA_clinicalMatrix.gz). A total of 933 specimens were used for this analysis as previously performed in other studies (add this ref: https://doi.org/10.1111/acel.13665). After ortholog mapping to convert murine genes to the human genes, the enrichment score of the original 79 genes signature was calculated for each patient using a single-sample gene set enrichment analysis (ssGSEA) from Gene Pattern 2.0 (ADD REF https://doi.org/10.1038/ng0506-500). Patients were then divided in high and low enrichment score using the top tertile. The differences in survival were represented using the Kaplan-Meier curve and log-rank test.

### Statistics

Statistical analyses were conducted using GraphPad Prism Software. A p-value cutoff of 0.05 was applied to determine significance levels. ns = not

significant, * p< 0.05, ** p< 0.01, *** p < 0.001, **** p < 0.0001.

Correlations of high and low signature patient groups with clinico-pathological characteristics were analyzed by Fisher’s exact test.
